# Supplementary material for: A porcine brain-wide RNA editing landscape
Source: Commun Biol. 2021 Jun 10;4:717. doi: 10.1038/s42003-021-02238-3 (PMC8192503; doi:10.1038/s42003-021-02238-3)
Supplement: Supplementary file 2 — Supplementary Information [file 42003_2021_2238_MOESM2_ESM.pdf]

Supplementary Figures for

A porcine brain-wide RNA editing landscape

Jinrong Huang, Lin Lin, Zhanying Dong, Ling Yang, Tianyu Zheng, Weiwang Gu, Yan Zhang, Tailang Yin, Evelina Sjöstedt, Jan Mulder, Mathias Uhlen, Karsten Kristiansen, Lars Bolund, Yonglun Luo

✉ To whom correspondence may be addressed: huangjinrong@genomics.cn (J.H.) or alun@biomed.au.dk (Y.L.)

Fig. S1

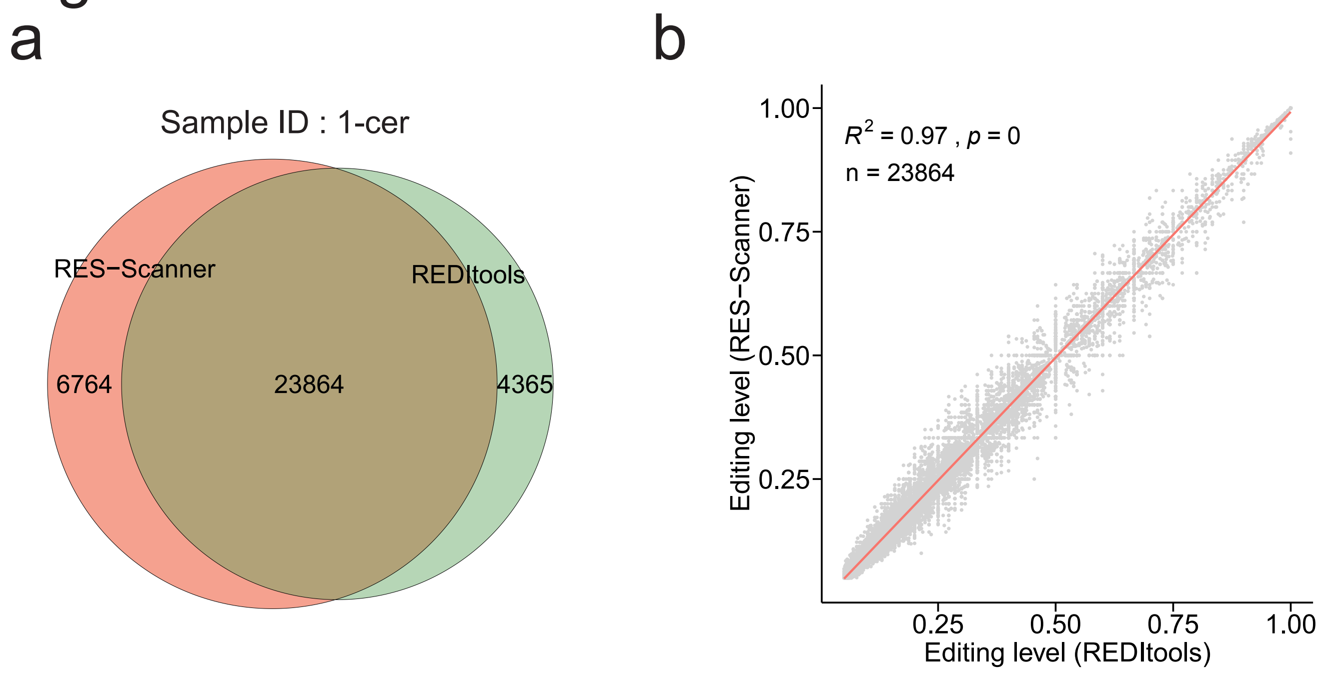

Fig. S1 | Comparison of results from REDItools and RES-Scanner. One sample (ID: 1-cer) from cerebellum is shown as an example. a, Venn diagram of RNA editing sites identified by two tools showing most of sites are overlapped. b, Correlations between editing levels of common sites identified by the two tools.

Fig. S2

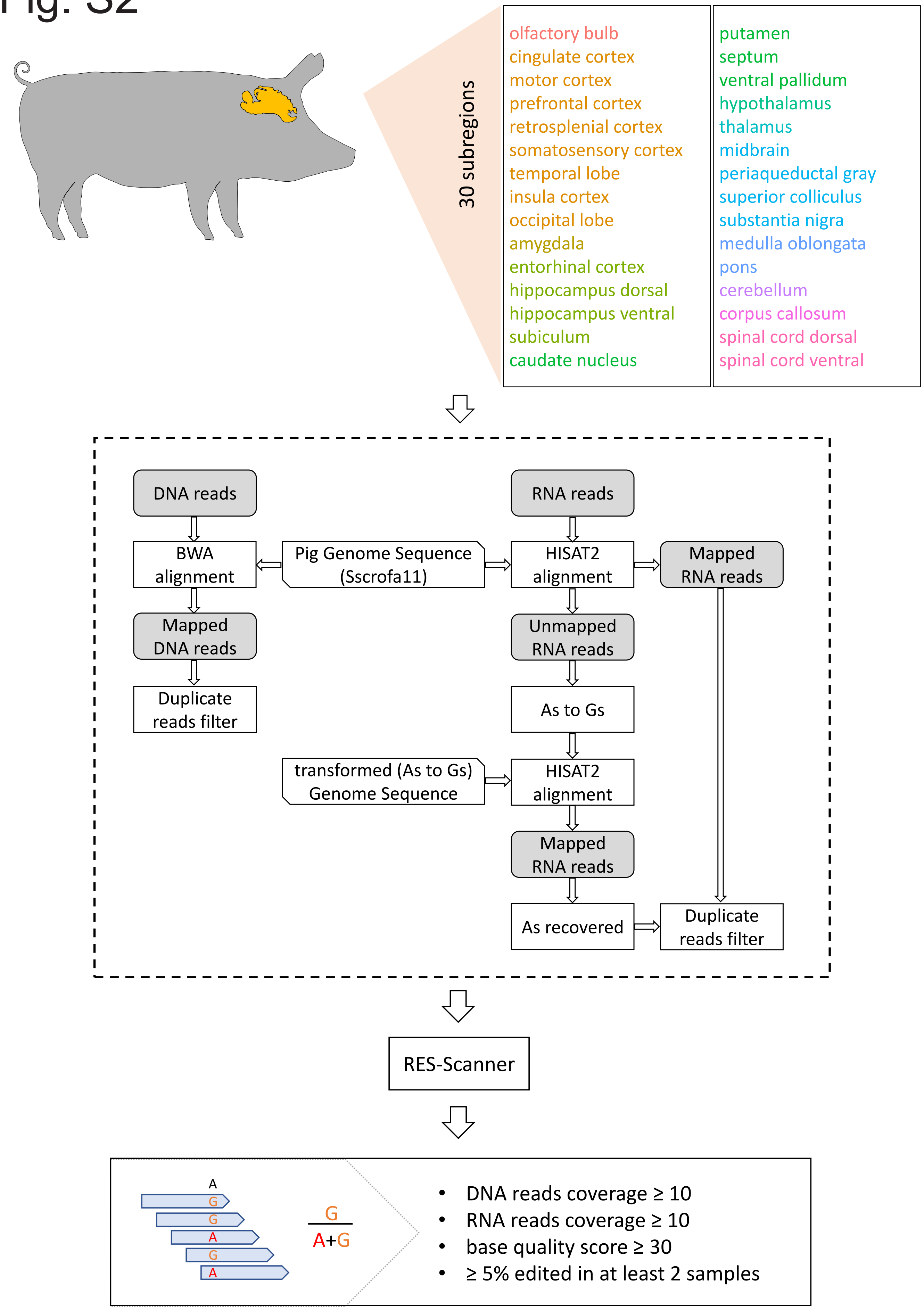

Fig. S2 | The pipeline of A-to-I RNA editing analysis. BWA (Burrows-Wheeler Aligner) is a program for mapping DNA reads against reference genome. HISAT2 (Hierarchical Indexing for Spliced Alignment of Transcripts) is a fast program for mapping RNA-seq reads against reference genome.

Fig. S3

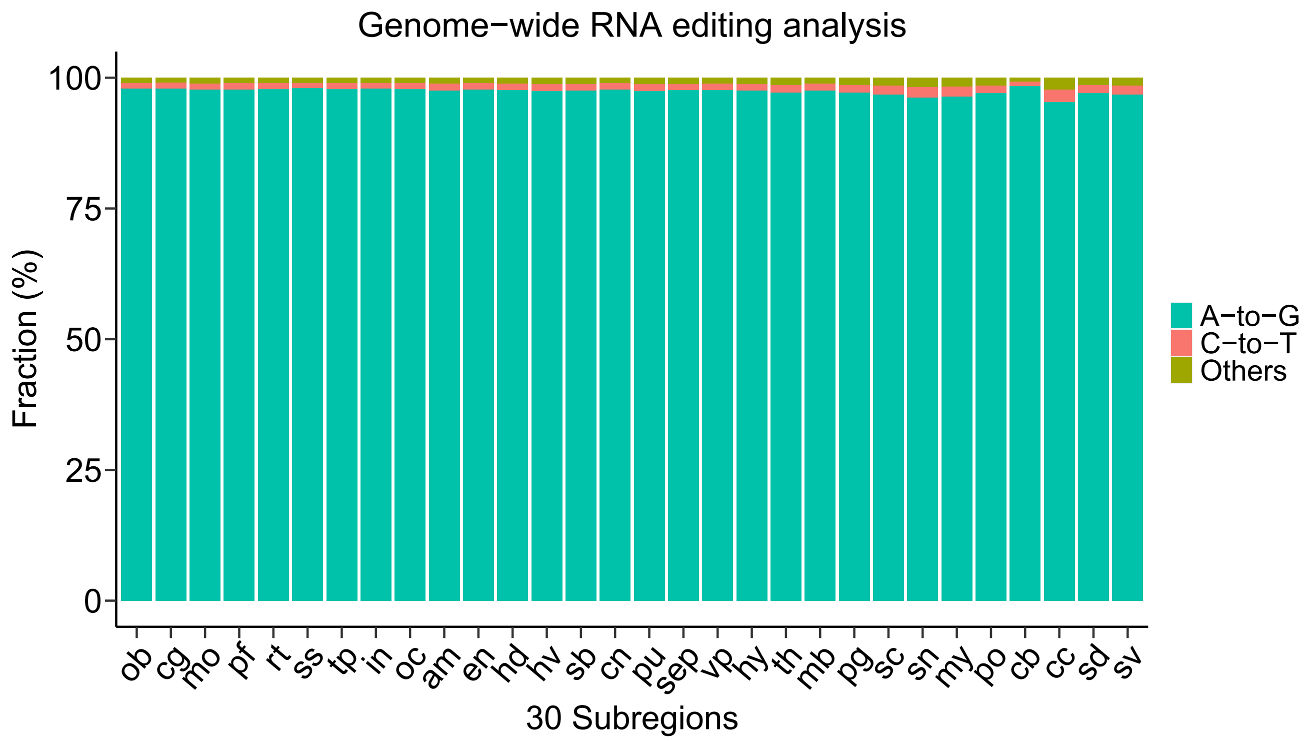

Fig. S3 | RNA editing type identified in 30 subregions of pig brain. olfactory bulb, ob; cingulate cortex, cg; motor cortex, mo; prefrontal cortex, pf; retrosplenial cortex, rt; somatosensory cortex, ss; temporal lobe, tp; insula cortex, in; occipital lobe, oc; amygdala, am; entorhinal cortex, en; hippocampus dorsal, hd; hippocampus ventral, hv; subiculum, sb; caudate nucleus, cn; putamen, pu; septum, sep; ventral pallidum, vp; hypothalamus, hy; thalamus, th; midbrain, mb; periaqueductal gray, pg; superior colliculus, sc; substantia nigra, sn; medulla oblongata, my; pons, po; cerebellum, cb; corpus callosum, cc; spinal cord dorsal, sd; spinal cord ventral, sv.

Fig. S4

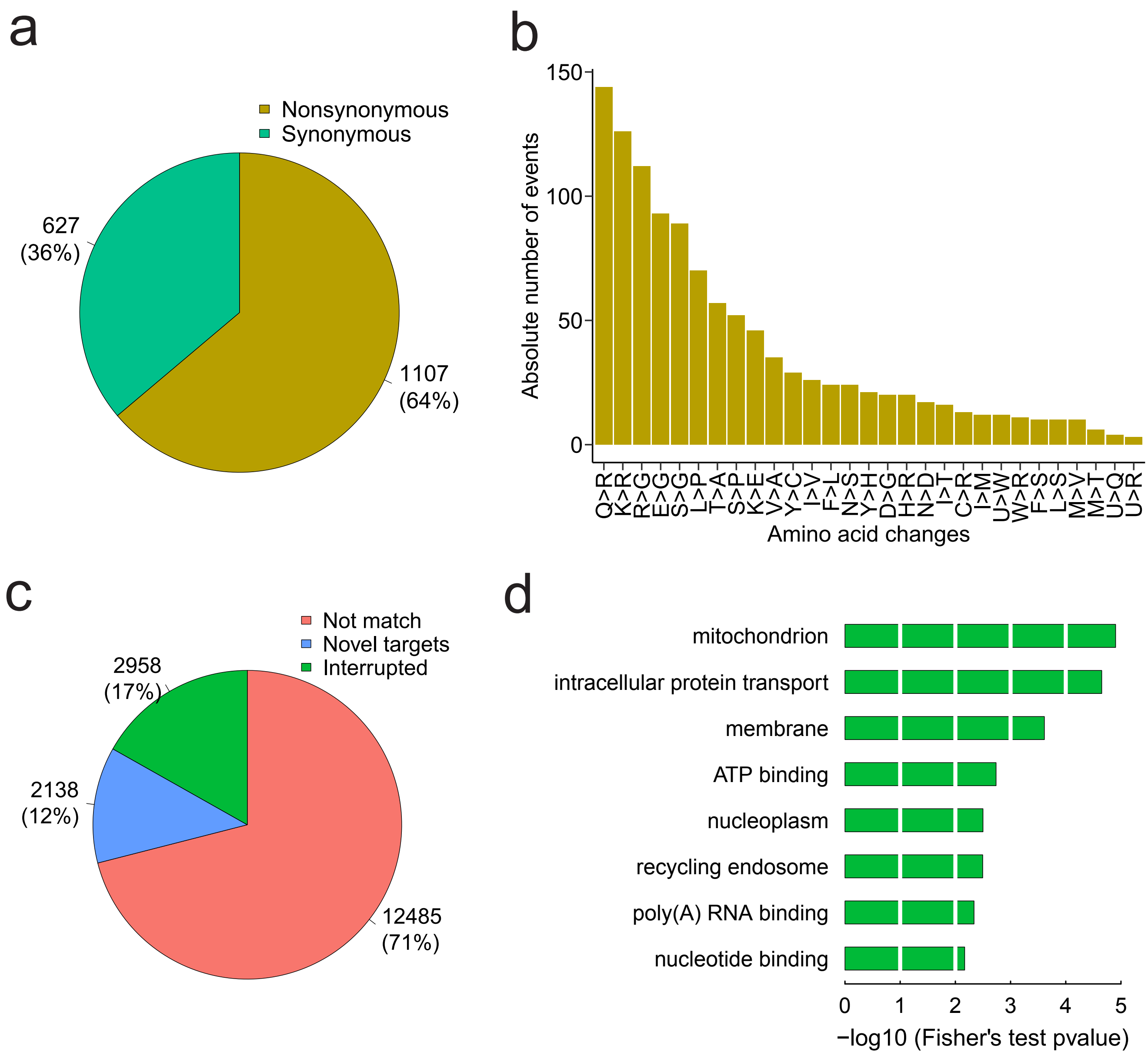

Fig. S4 | The A-to-I editing sites located in CDS and 3-UTRs. a, The number of nonsynonymous (recoding) and synonymous editing sites. b, Distribution of amino acid changes type. c, Distribution of editing sites relative to miRNA target sites in 3-UTRs. Not match, editing sites without miRNA target; Novel targets, editing sites which potentially create novel miRNA target; Interrupted, editing sites which potentially interrupt miRNA target. d, The top Gene ontology (GO) terms associated with genes harboring editing sites which potentially interrupt miRNA target.

Fig. S5

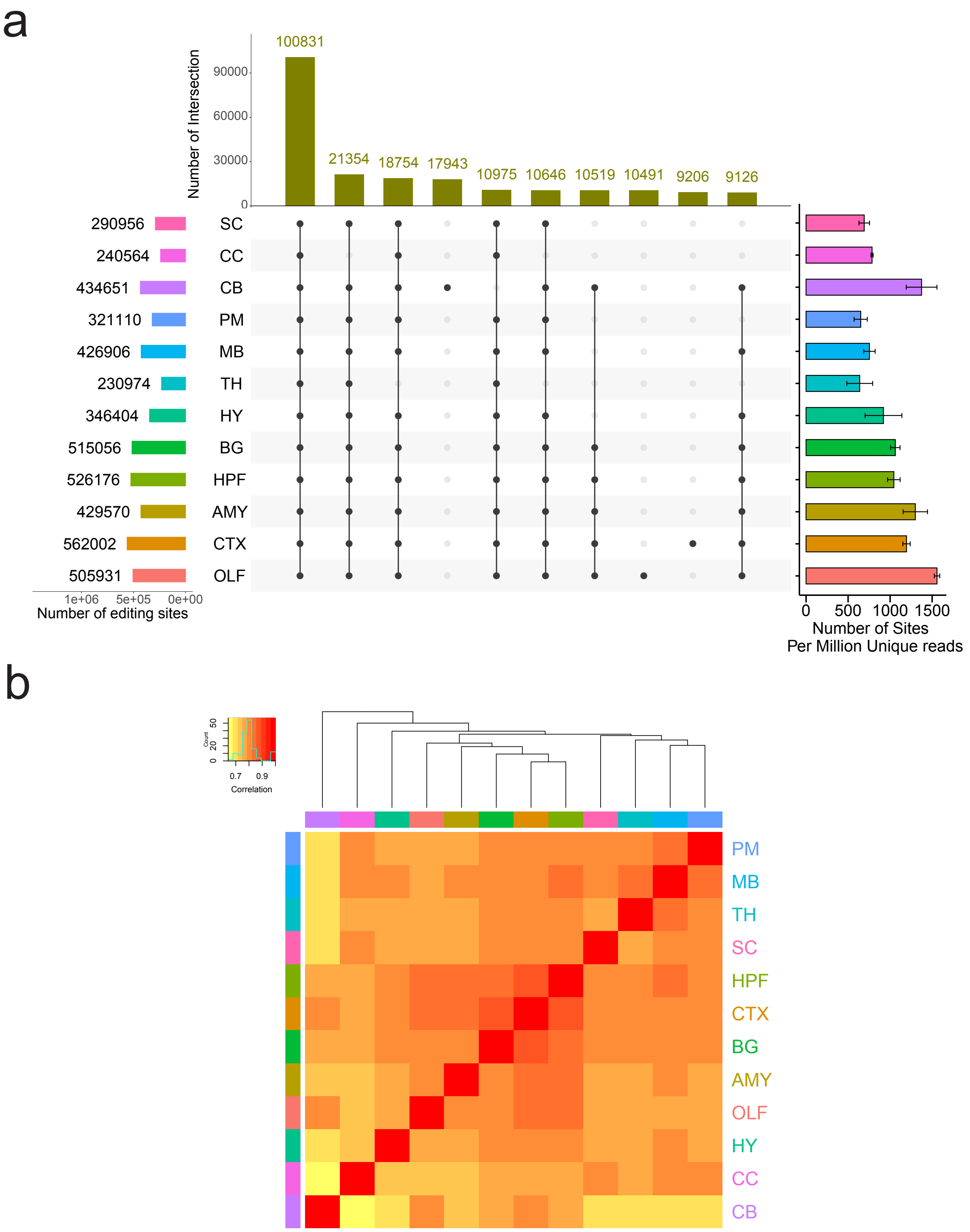

Fig. S5 | Landscape of RNA editing sites across pig brain regions. a, UpSetR plot showing the number of editing sites identified across 12 brain regions and the top ten intersection among regions. The number of A-to-I editing sites were normalized by uniquely mapped reads each sample on the right. b, Heatmap and dendrogram of correlations on the editing levels of different regions.

Fig. S6

a

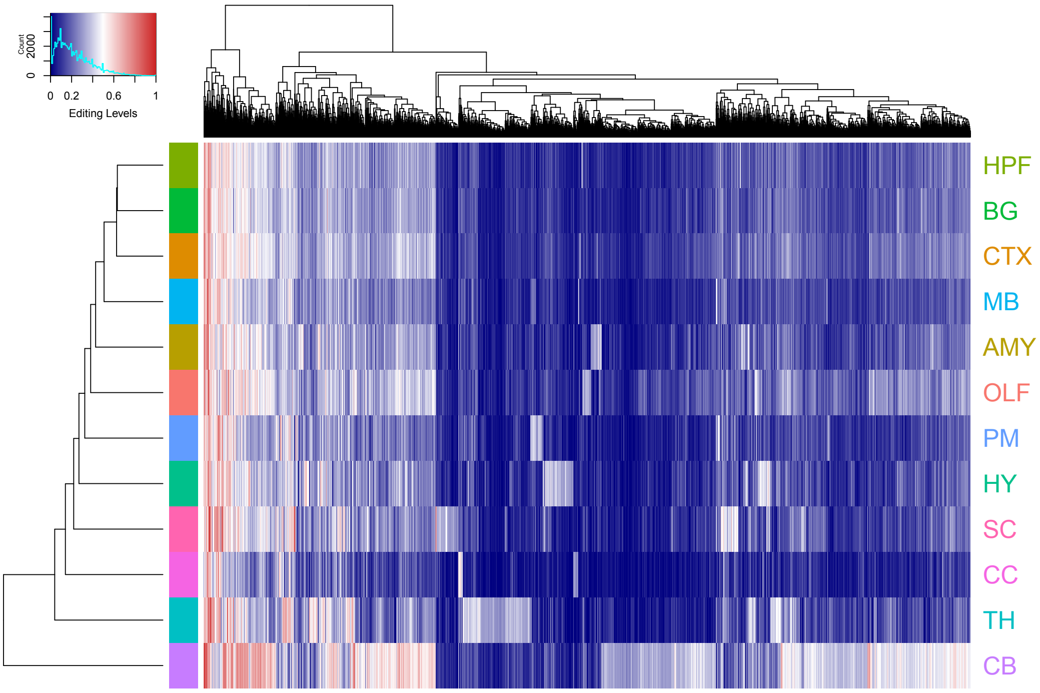

b

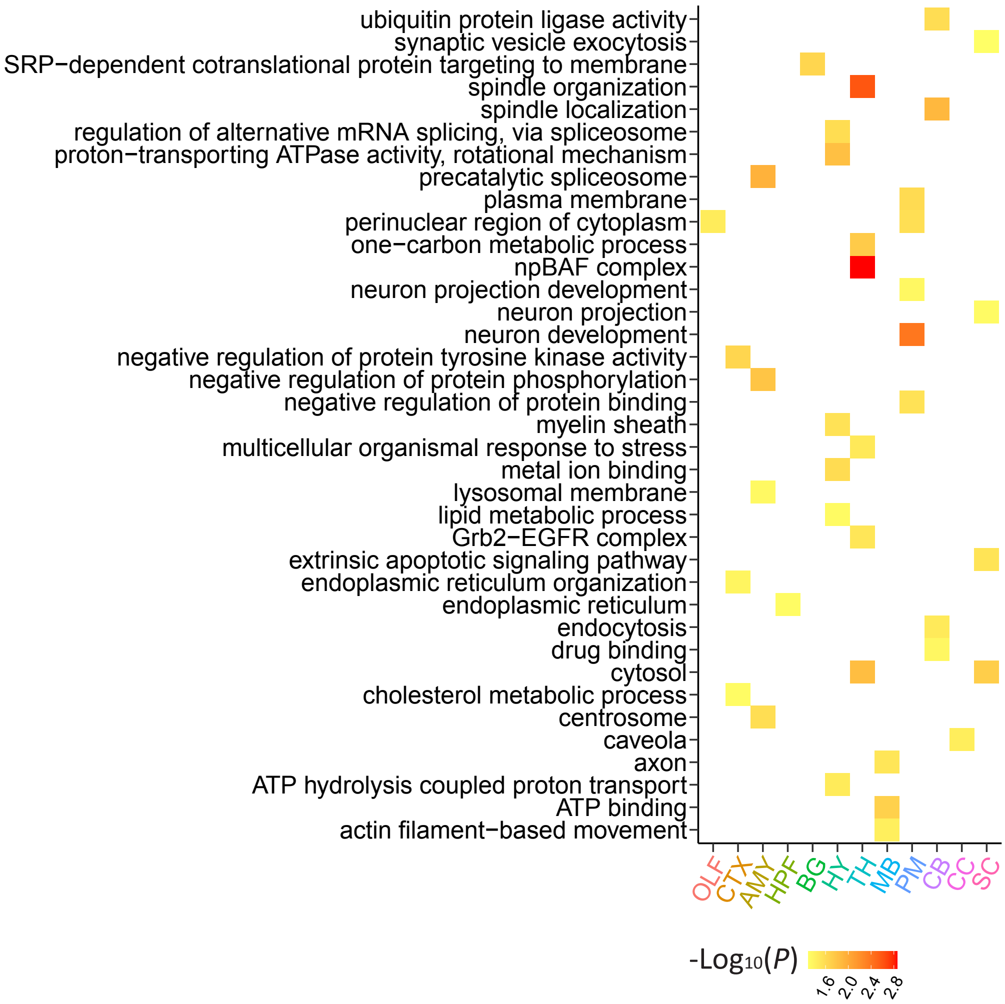

Fig. S6 | Region-specific editing sites within the pig brain. a, Heatmap showing region-specific editing sites with coverage ≥ 10 RNA reads in all regions. b, Go enrichment analysis of genes harboring at least one region-enriched editing site.

Fig. S7

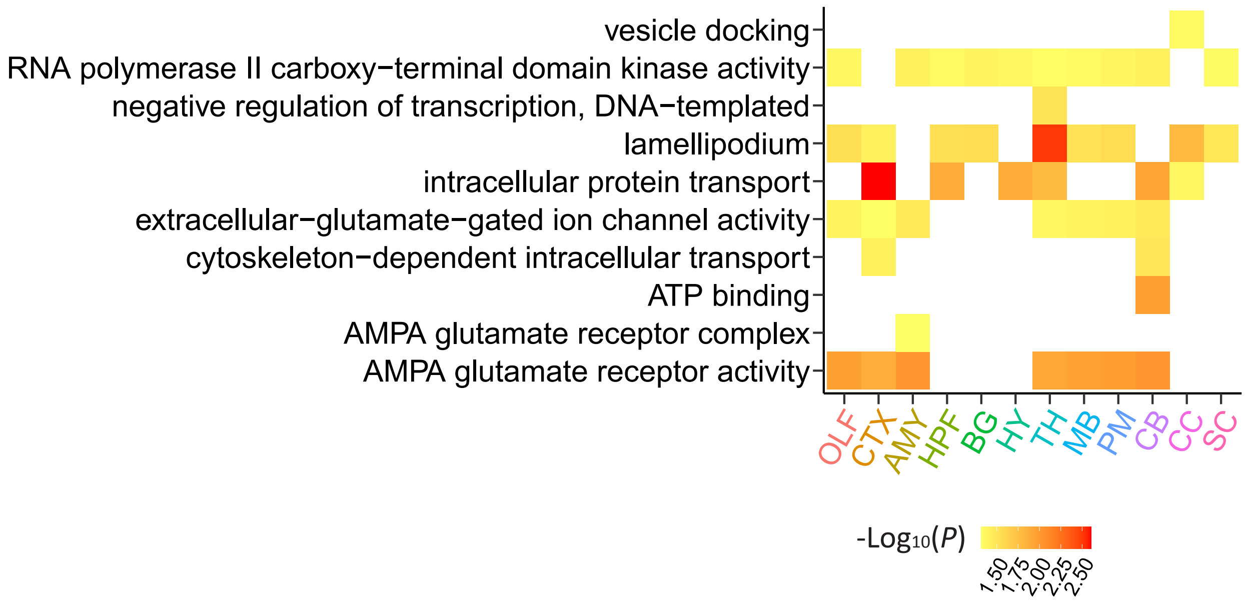

Fig. S7 | Go enrichment analysis of genes harboring highly edited (>75%) CDS or 3-UTR residing sites

Fig. S8

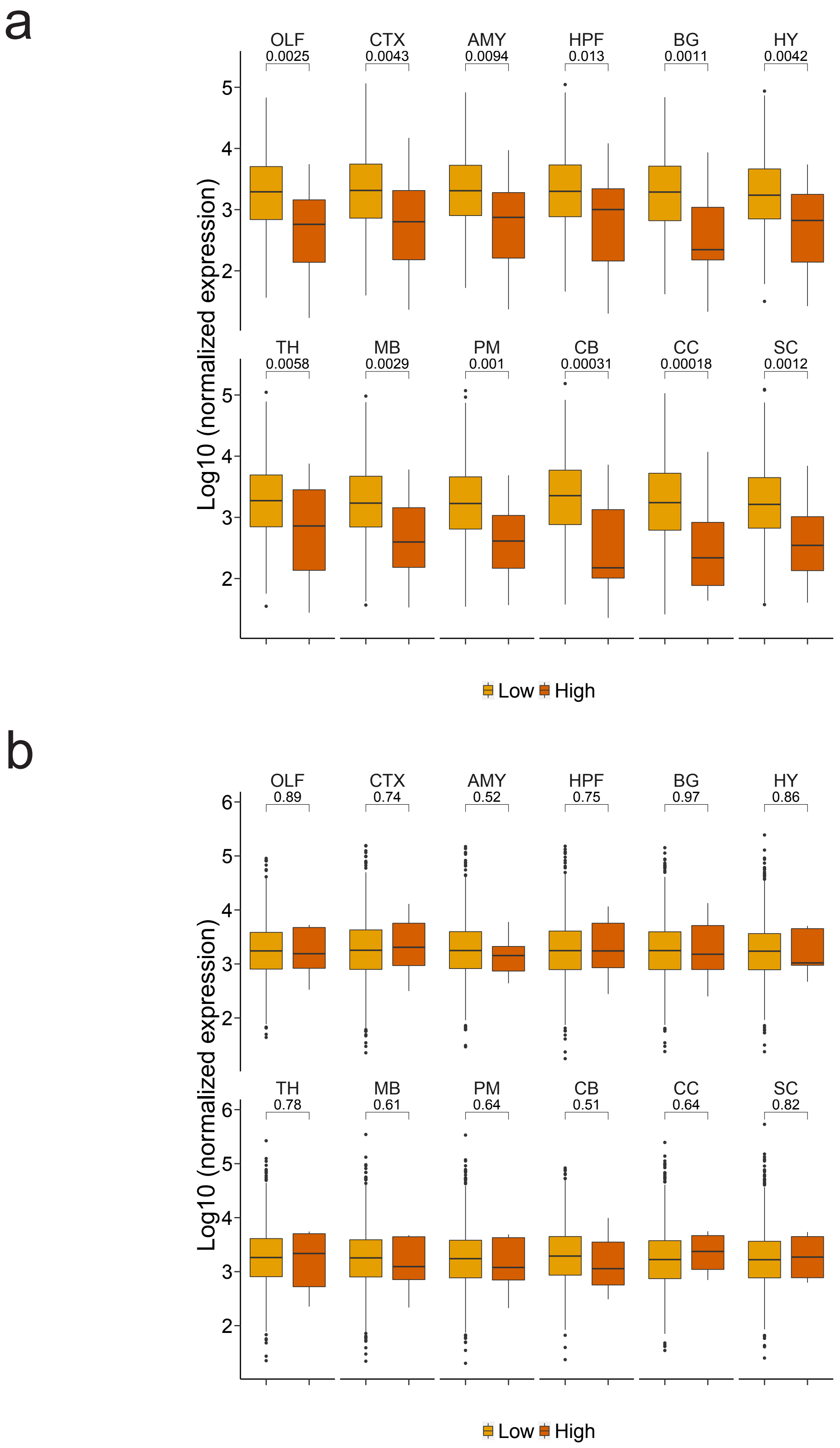

Fig. S8 | Box plots showing expression level of genes with highly and lowly edited sites. a, CDS-residing sites. b, 3-UTR residing sites. The significance of differences in expression was assessed by Wilcoxon test.

Fig. S9

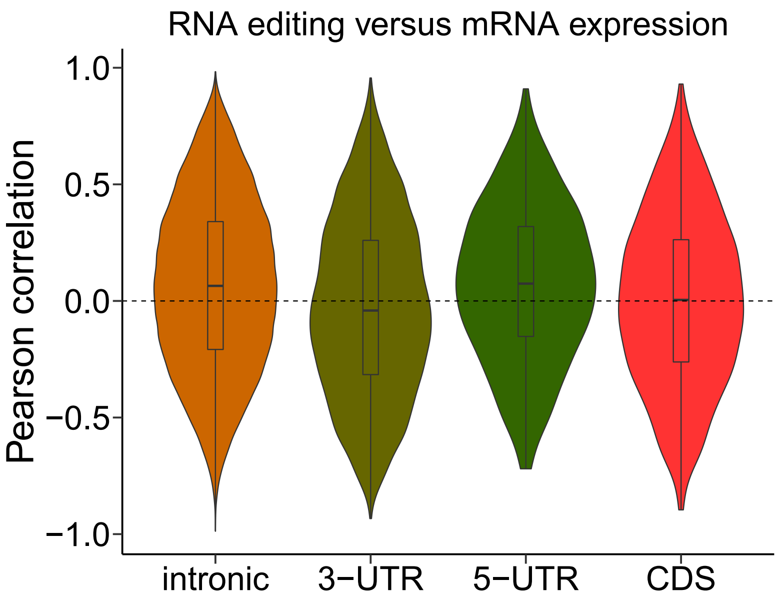

Fig. S9 | Pearson correlation between RNA editing and mRNA expression.

Fig. S10

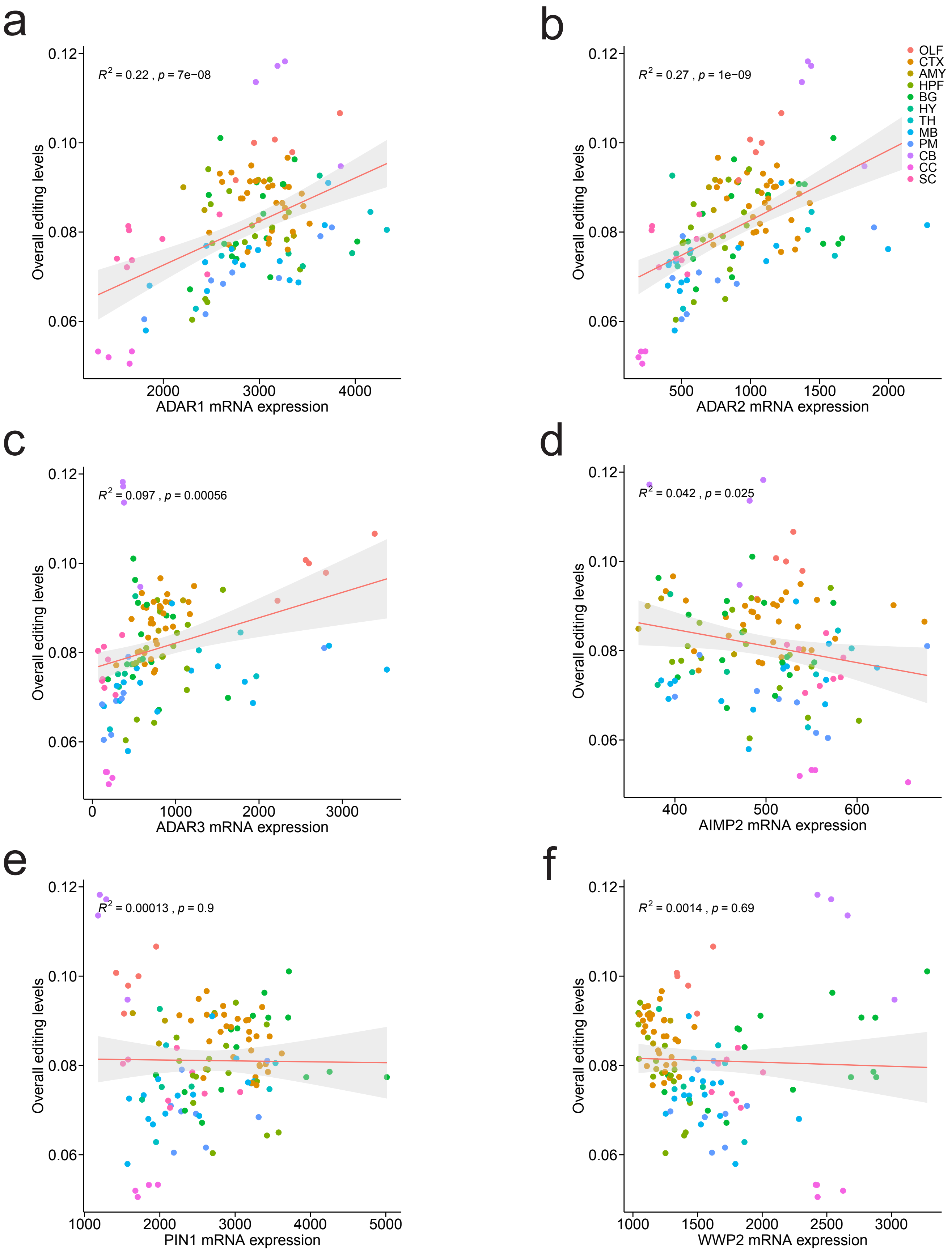

Fig. S10 | Known enzymes associated with A-to-I RNA editing. Correlations between overall editing levels at all editing sites and mRNA expression levels of ADAR1 (a), ADAR2 (b), ADAR3 (c), AIMP2 (d), PIN1 (e) and WWP2 (f). R^2 values were calculated by robust linear regressions on overall editing levels and normalized expression. Gray shaded areas, 95% confidence interval.

Fig. S11

a

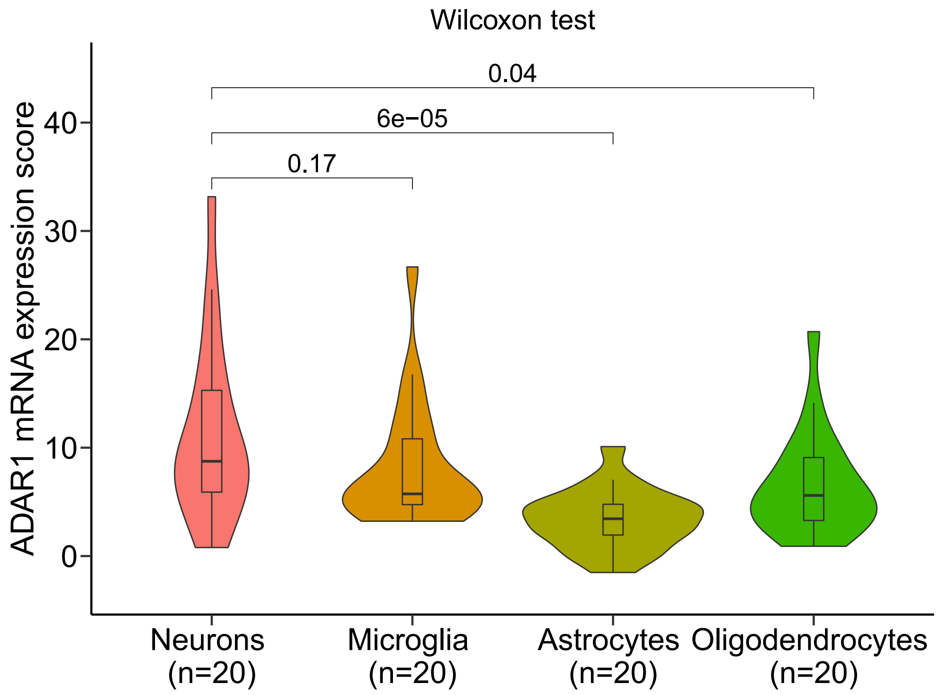

b

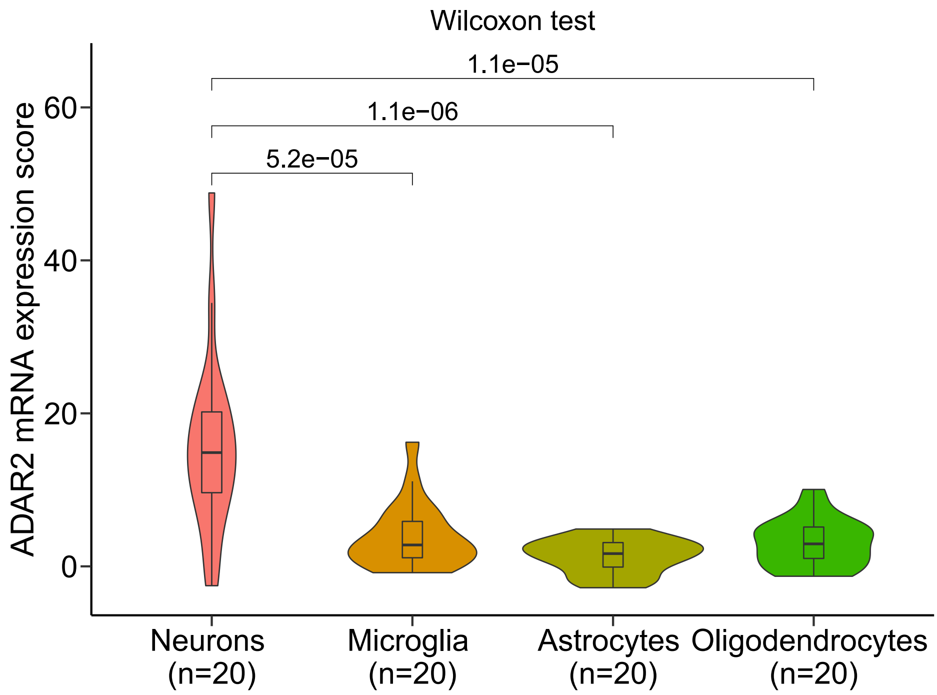

Fig. S11 | The ADARs mRNA expression scores of neurons, microglia, astrocytes and oligodendrocytes. a, ADAR1. b, ADAR2. The significance of differences between expression scores of ADAR1 or ADAR2 in neurons and non-neuronal cells was assessed by Wilcoxon test.

Fig. S12

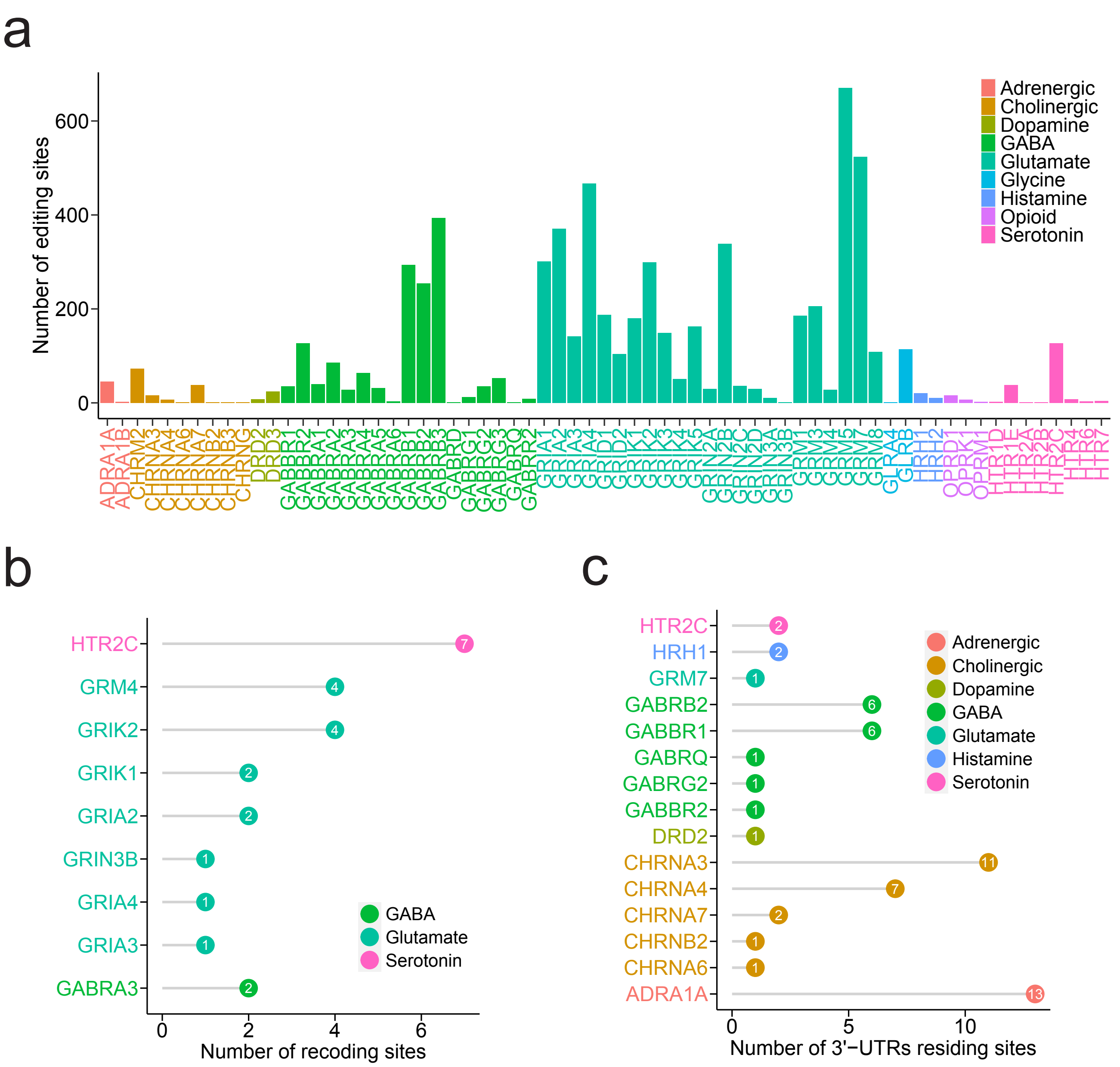

Fig. S12 | Distribution of the A-to-I RNA editing sites located in major neurotransmitter receptors. a, RNA editing sites located in neurotransmitter receptors, including adrenergic, cholinergic, dopamine, GABA, glutamate, glycine, histamine, opioid and serotonin receptors. b, The number of recoding editing sites. c, The number of editing sites located in 3-UTRs.

Fig. S13

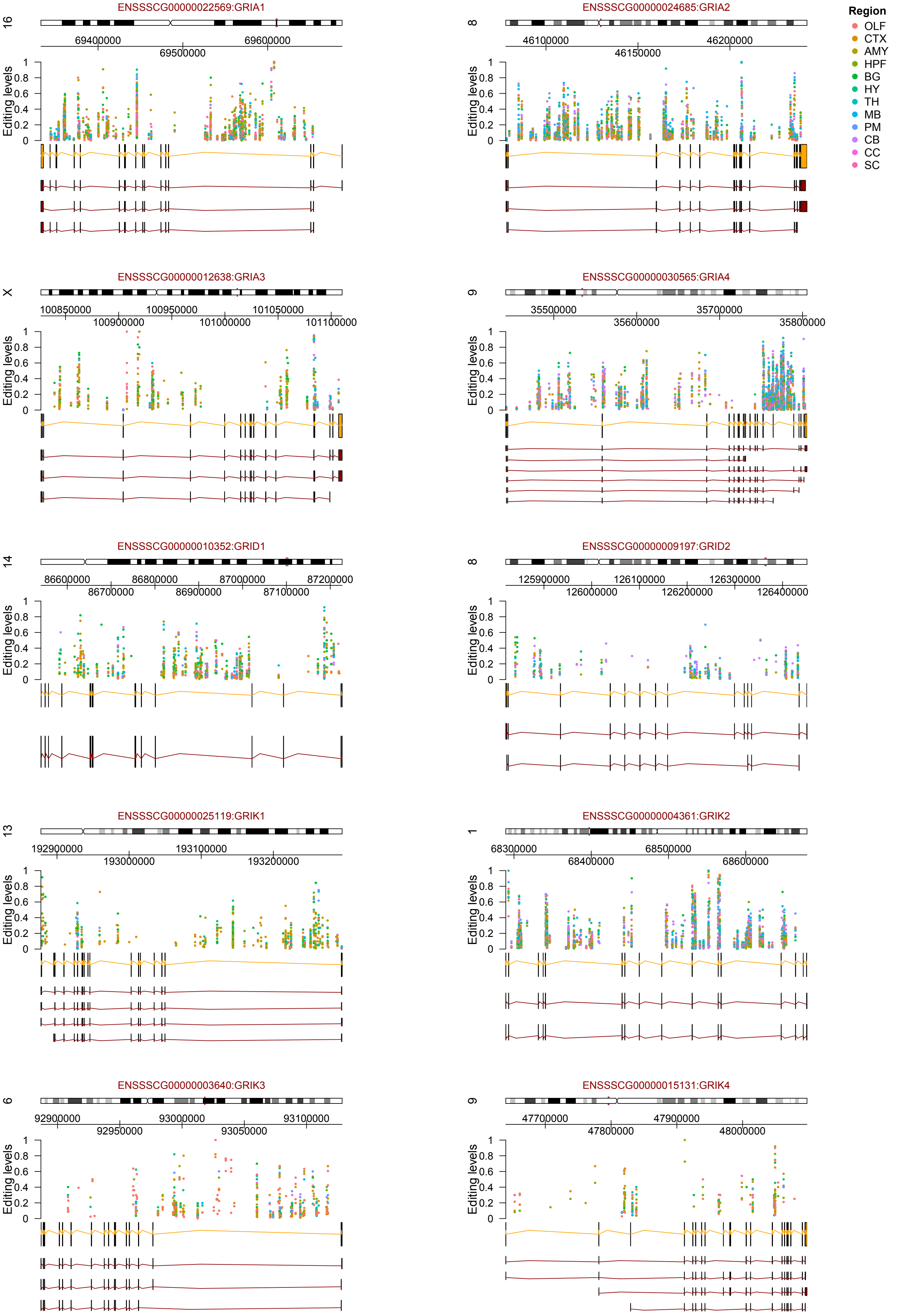

ENSSSCG00000022569:GRIA1
ENSSSCG00000024685:GRIA2
ENSSSCG00000012638:GRIA3
ENSSSCG00000030565:GRIA4
ENSSSCG00000010352:GRID1
ENSSSCG00000009197:GRID2
ENSSSCG00000025119:GRIK1
ENSSSCG00000004361:GRIK2
ENSSSCG00000003640:GRIK3
ENSSSCG00000015131:GRIK4
Editing levels
Region
OLF
CTX
AMY
HPF
BG
HY
TH
MB
PM
CB
CC
SC

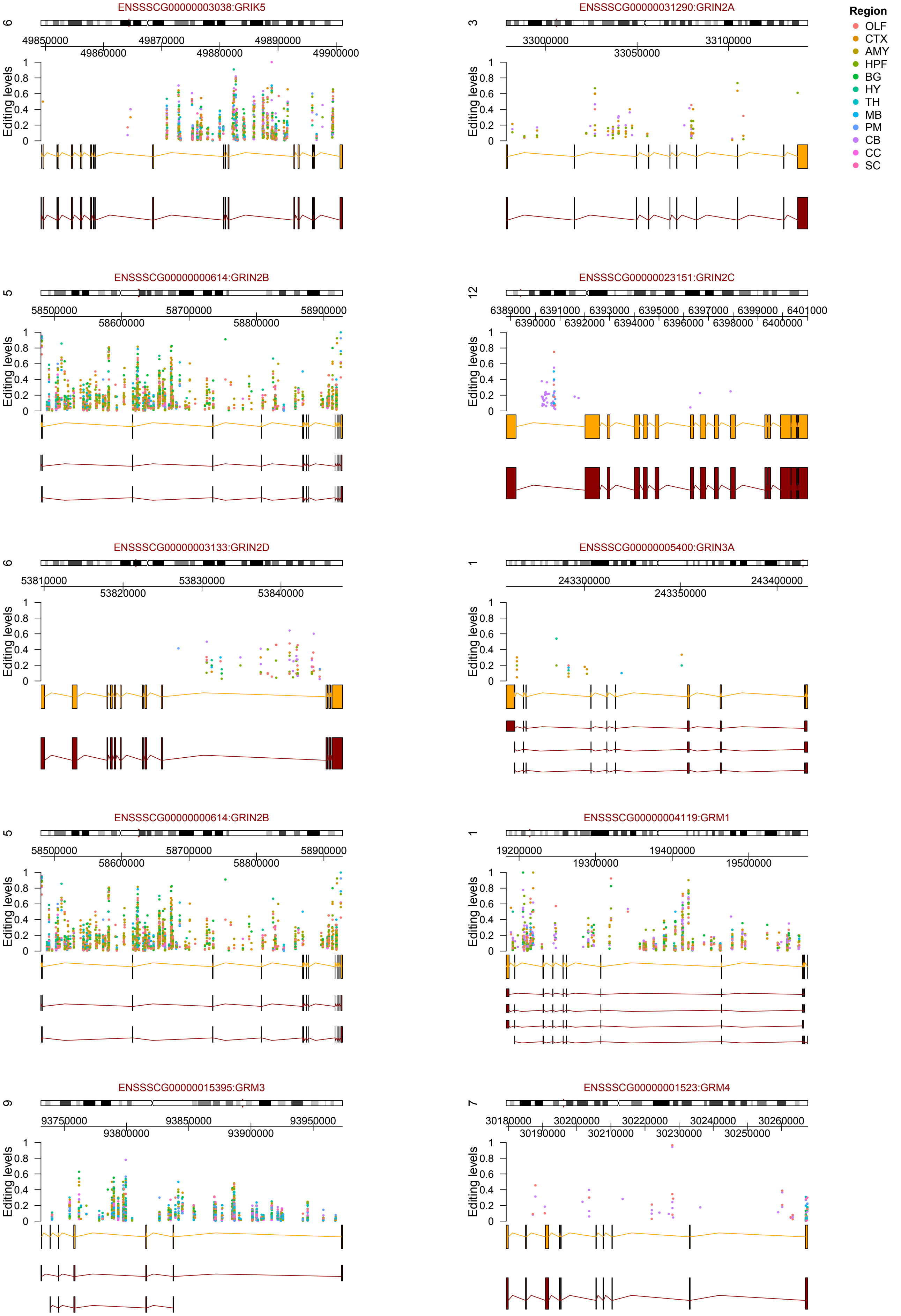

Region
OLF
CTX
AMY
HPF
BG
HY
TH
MB
PM
CB
CC
SC
ENSSSCG00000003038:GRIK5
6
49850000
49860000
49870000
49880000
49890000
49900000
Editing levels
0
0.2
0.4
0.6
0.8
1
ENSSSCG00000031290:GRIN2A
3
33000000
33050000
33100000
ENSSSCG00000000614:GRIN2B
5
58500000
58600000
58700000
58800000
58900000
ENSSSCG00000023151:GRIN2C
12
6389000
6390000
6391000
6392000
6393000
6394000
6395000
6396000
6397000
6398000
6399000
6400000
6401000
ENSSSCG00000003133:GRIN2D
6
53810000
53820000
53830000
53840000
ENSSSCG00000005400:GRIN3A
1
243300000
243350000
243400000
ENSSSCG00000000614:GRIN2B
5
58500000
58600000
58700000
58800000
58900000
ENSSSCG00000004119:GRM1
1
19200000
19300000
19400000
19500000
ENSSSCG00000015395:GRM3
9
93750000
93800000
93850000
93900000
93950000
ENSSSCG00000001523:GRM4
7
30180000
30190000
30200000
30210000
30220000
30230000
30240000
30250000
30260000

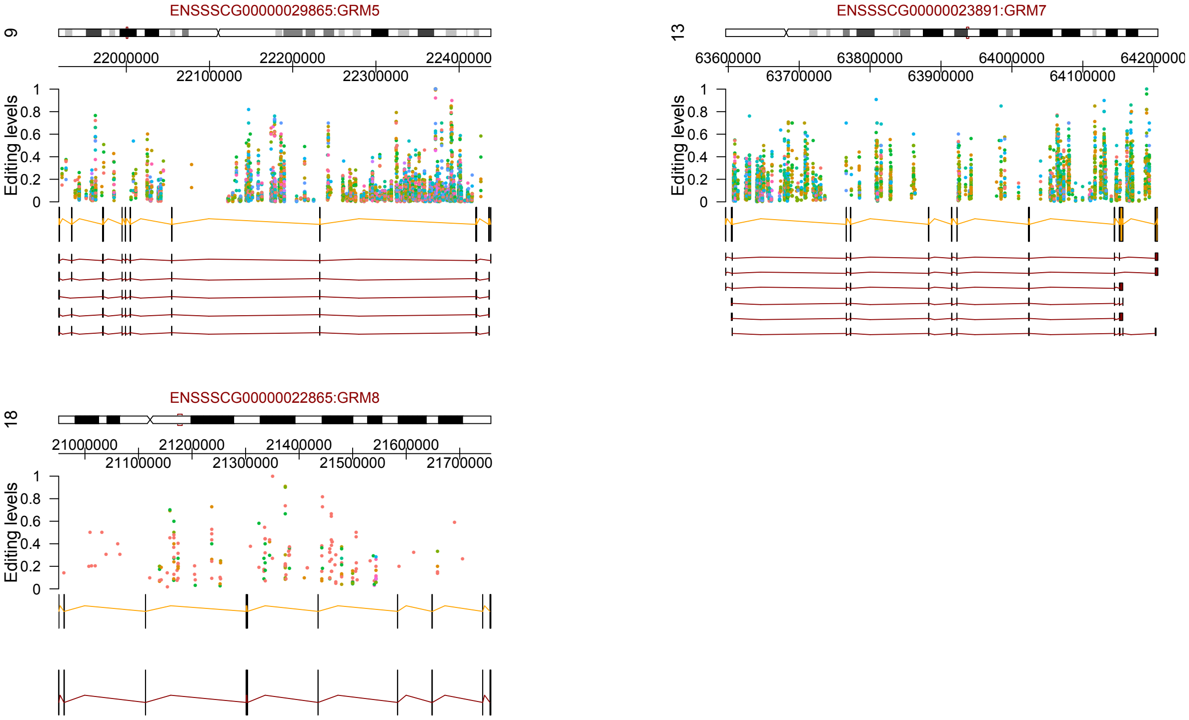

Fig. S13 | Detailed information of editing sites located in glutamate receptors. Olfactory bulb, OLF; Cerebral cortex, CTX; Amygdala, AMY; Hippocampal formation, HPF; Basal ganglia, BG; Hypothalamus, HY; Thalamus, TH; Midbrain, MB; Pons and medulla, PM; Cerebellum, CB; Corpus callosum, CC; Spinal cord, SC.

Fig. S14

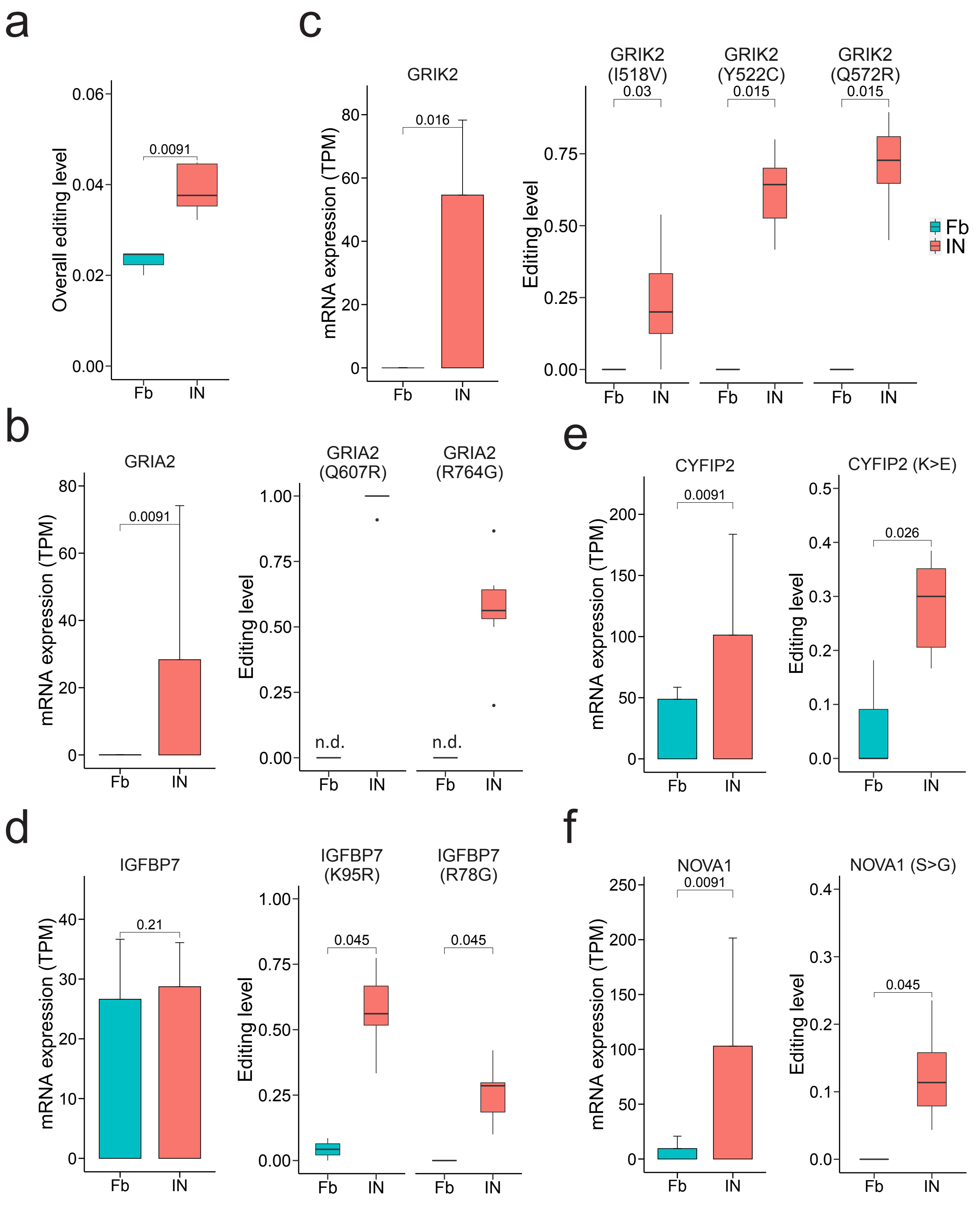

Fig. S14 | Increased editing from fibroblasts to induced neurons. a, Overall editing level of fibroblasts and neurons. The mRNA expression and recoding events of GRIA2 (b), GRIK2 (c), IGFBP7 (d), CYFIP2 (e) and NOVA1 (f). Not detected, n.d.; Fibroblasts, Fb; induced neurons, IN. The significance of differences was assessed by Wilcoxon test.

Fig. S15

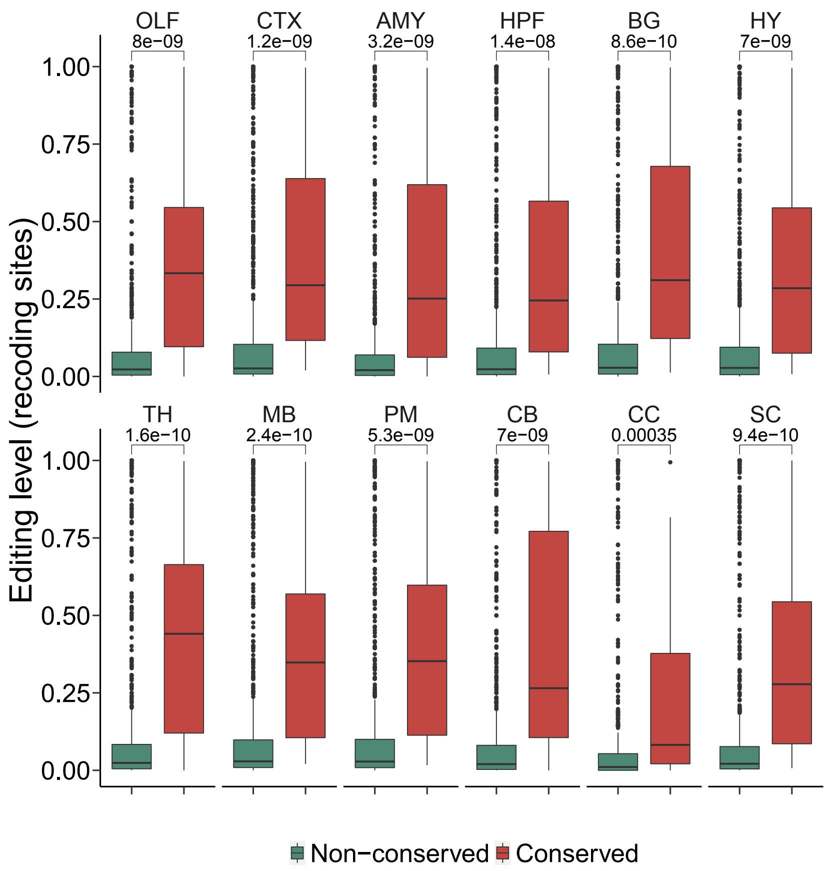

Fig. S15 | Box plot showing editing level of conserved and non-conserved recoding sites. The significance of differences was assessed by Wilcoxon test.

Fig. S16

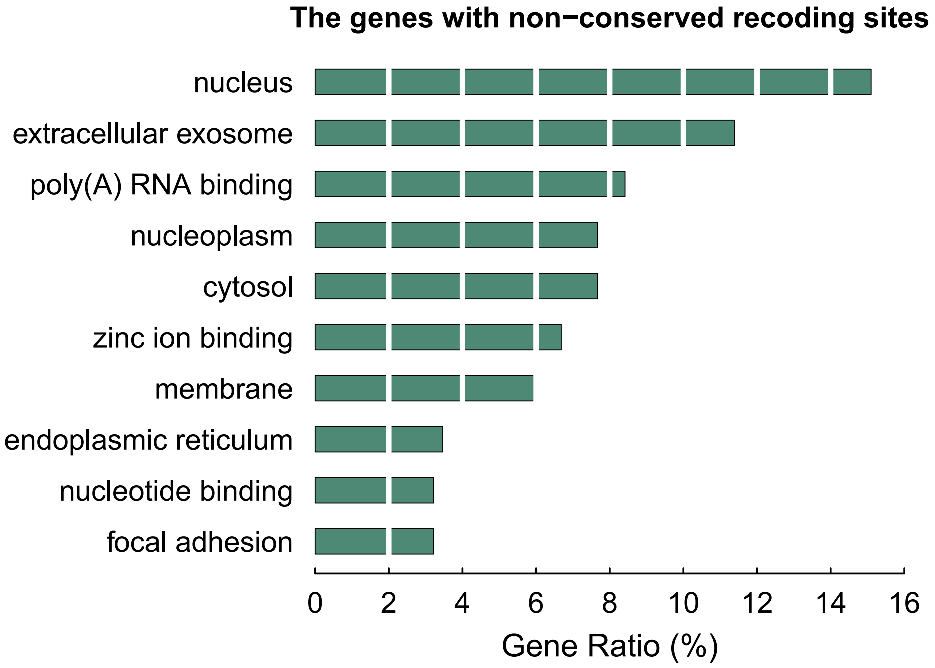

Fig. S16 | Bar plot showing categories of genes with non-conserved recoding sites. Only the top ten categories are shown (ranked by gene ratio).

Fig. S17

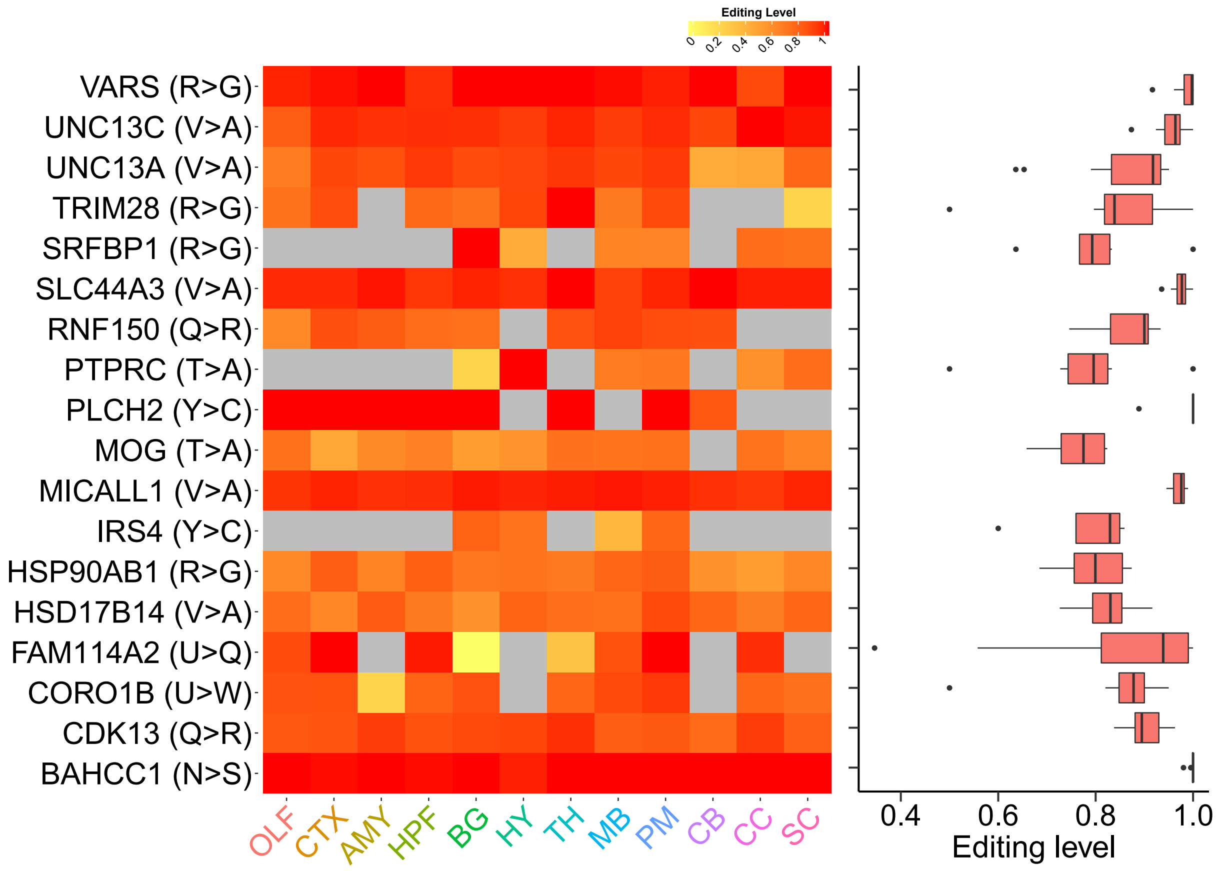

Fig. S17 | The highly edited non-conserved recoding sites in pig brain. The editing sites were > 75% edited in at least one brain region.

Fig. S18

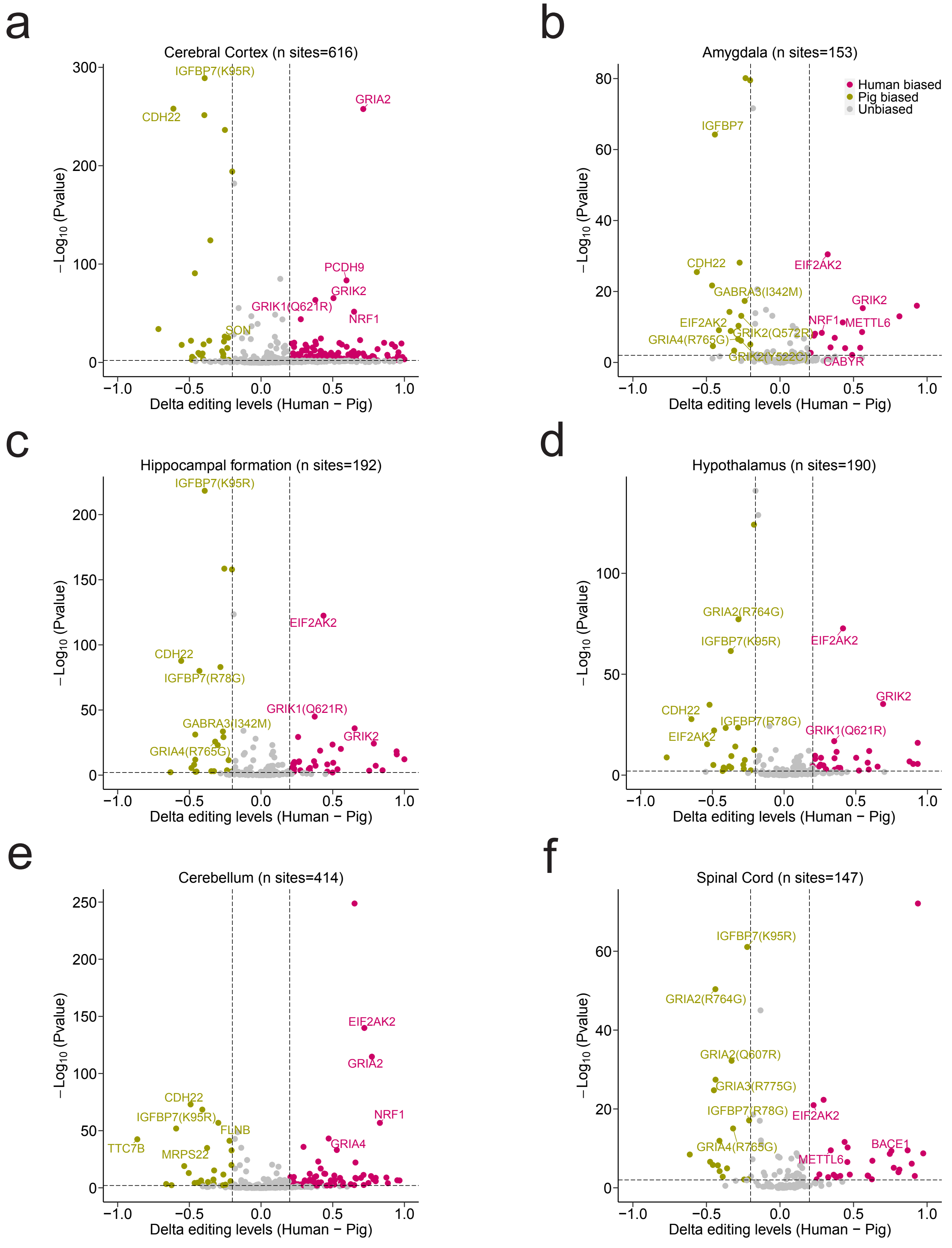

Fig. S18 | Volcano plots showing species-biased editing sites in pig and human brain. (a) cerebral cortex, (b) amygdala, (c) hippocampal formation, (d) hypothalamus, (e) cerebellum and (f) spinal cord. The X-axis represents the differential editing levels between pig and human. The Y-axis represents negative $\log_{10}$ (Fisher's exact test p-value). For top differentially editing sites in genic region, their respective orthologous genes were indicated.

Fig. S19

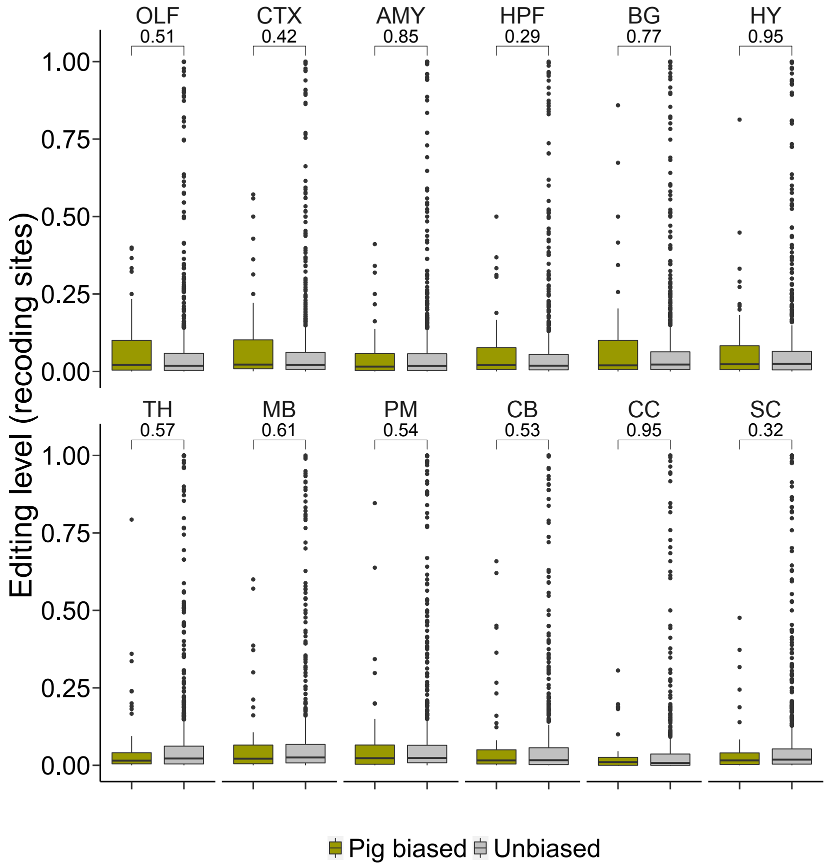

Fig. S19 | Box plot showing editing level of recoding sites located in genes showing pig-biased or unbiased expression. The significance of differences was assessed by Wilcoxon test.
