# Supplementary material for: A porcine brain-wide RNA editing landscape
Source: Commun Biol. 2021 Jun 10;4:717. doi: 10.1038/s42003-021-02238-3 (PMC8192503; doi:10.1038/s42003-021-02238-3)
Supplement: Supplementary file 3 — Description of Supplementary Files [file 42003_2021_2238_MOESM3_ESM.pdf]

Description of Additional Supplementary Files

File name: Supplementary Data

Description:

Supplementary Data 1: List of all the 119 pig brain samples included in the analysis.

Supplementary Data 2: Sequencing information of whole genome sequencing data

Supplementary Data 3a: List of conserved editing sites in Cerebral Cortex.

Supplementary Data 3b: List of conserved editing sites in Amygdala.

Supplementary Data 3c: List of conserved editing sites in Hippocampal formation.

Supplementary Data 3d: List of conserved editing sites in Hypothalamus.

Supplementary Data 3e: List of conserved editing sites in Cerebellum.

Supplementary Data 3f: List of conserved editing sites in Spinal Cord.

Supplementary Data 4: List of samples obtained from GTEx Portal.
